# The Adaptation of Participation Scale Short Simplified Questionnaire into Indonesian Language and the Psychometric Properties in Individuals with Type 2 Diabetes Mellitus with Vestibular Dysfunction

**DOI:** 10.1155/2022/2565833

**Published:** 2022-06-15

**Authors:** Dwi Rosella Komalasari, Mantana Vongsirinavarat, Vimonwan Hiengkaew, Nantinee Nualnim

**Affiliations:** Faculty of Physical Therapy, Mahidol University, 999, Salaya, Phuttamonthon, Nakhon Pathom 73170, Thailand

## Abstract

**Background:**

Type 2 diabetes mellitus (T2DM) has been reported to affect the vestibular system resulting in dizziness and vertigo complaints. This complication is known to disable the social participation. The Participation Scale Short Simplified (PSSS) has been developed to quantify the severity of social participation restrictions. The aim of this study was to translate and cross-culturally adapt the PSSS into Indonesian Bahasa (PSSS-Ina). The measurement properties of the translated version and the factors contributing to the severe participation restriction were determined.

**Methods:**

The participants comprised 55 T2DM with vestibular dysfunction (VD) in the community center for diabetes mellitus in Central Java, Indonesia. The signs of VD were confirmed by head impulse test, Dix Hallpike Test, and supine roll test. The PSSS-Ina was administered twice with a four-week interval. The physical examination was also performed to identify the contributing factors.

**Results:**

The test–retest reliability of the PSSS Indonesian Bahasa version (PSSS-Ina) between two measurement sessions was excellent (ICC of 0.93, *p* < 0.001, and 95% CI: 0.88–0.95). The correlation coefficient between two administrations was high (*r* = 0.88). Based on the demonstrated content validity, the values of the corrected item and total correlation were greater than 0.3. No floor and ceiling effects were observed. The good internal consistency was confirmed with Cronbach's alpha of 0.84. The factor analysis produced three factors of activity participation, social engagement, and work-related participation. The multiple logistic regression revealed that the balance performance of mCTSIB was the main factor contributing to the severe participation restriction reflected by the PSSS-Ina score.

**Conclusion:**

The Indonesian version of the PSSS-Ina demonstrated excellent comprehensibility and reliability in individuals suffering T2DM with VD. This tool is therefore helpful in identifying the participation limitation in individuals with VD.

## 1. Introduction

Diabetes mellitus (DM) is a prevalent metabolic condition affecting the ability in regulating blood glucose level. Specifically, type 2 DM (T2DM) is known to be directly linked to the lifestyle factors and becomes a key health issue due to various complications [1]. Some of the common complications such as the peripheral neuropathy and retinopathy are well documented in their contributions to falling [2] and fear of falling [3] and significantly degrade the quality of life [1]. Moreover, these complications were reportedly impacting increased disability, lost productivity, mortality, and amplified health-care costs [4].

Vestibular dysfunction (VD) is another prevalent microvascular complication of T2DM which needs more recognition [5]. The physiological changes of peripheral vestibular apparatus causing dizziness and vertigo symptoms were widely observed in persons with diabetes [6]. The result of an epidemiological study showed that the VD occurred in up to 70% of patients with T2DM [7]. The persons older than 40 years and who suffered with T2DM more than 5 years who had dizziness symptom were 12 times more likely to report fall and risks of falling [8]. The vertigo symptom associated with the benign paroxysmal positional vertigo (BPPV) condition was also reportedly higher among patients with DM compared with the group without DM [9]. Dizziness and vertigo symptom is also known to compromise daily activities and functional status [10]. A study showed that 80% of subjects with VD experienced dizziness symptom which might be a predictor of imbalance and falls [7]. This symptom was also associated with fear of falling, falls, depression or anxiety, lower quality of life, and general health [11, 12], as well as reduced participation [13]. The dizziness in DM patients is influenced by many factors, both the VD itself and the side effects of diabetes medications [14]. Therefore, this disabling symptom needs early detection which can be preliminary confirmed by the clinical tests such as the Head Impulse Test and the Dix Hallpike test [15].

The increase of disability was evident among patients with T2DM who have dizziness complaint [16, 17]. However, the issue of social participation in these patients has not been extensively explored. Providing specific monitoring, the participation scale (P-scale) is the tool aiming to measure participation in rehabilitation, stigma reduction, and social integration programs. Based on the ICF model, P-scale consists of 18 items asking about the participants' abilities compared to their peer and their experiences to participate in various aspects of life [18]. With some comments on the original version [18], the P-scale was modified to be more applicable in low- and middle-income countries, as well as for monitoring the impact of interventions aimed at reducing disabilities affecting social participation [19].

The P-scale has been translated into many languages and demonstrates high internal consistency (Cronbach's alpha = 0.92), high interrater reliability (*r* = 0.80), and high discriminant validity. It has been used in different target groups, such as people with leprosy in Indonesia and AIDS in Nepal, India, and Brazil [18]. The questionnaire was then modified to 15 items [20]. Finally, the version of 13-item of Participation Scale Short (PSS) was tested and proofed for its applicability [21, 22]. The PSS in Nepali version was reported to have good sensitivity and specificity of 0.88 and 0.80, respectively, and good validity [23]. However, the scale format and the structure of questions were still problematic and further revision was suggested [24]. Coltof et al. was therefore recently revised and simplified the PSS to the Participation Scale Short Simplified (PSSS) for assessing participation restriction among persons affected by leprosy in Indonesia [24]. Good sensitivity (0.82) and specificity (0.75), internal consistency (Cronbach's alpha = 0.84), and reproducibility (ICC = 0.74) were reported [24]. However, this scale was perceived difficult to understand for people with low education [24].

T2DM is considered a tremendous challenge to the healthcare system in Indonesia with 7^th^ rank in the world, and about 10.7 million people suffered [25]. Although the disability induced by this condition is well recognized, its contributions to social life have not been fully explored. Very few studies have reported the social participation of persons with T2DM with vestibular dysfunction [26]. Although the available Indonesian version of PSSS scale was reported to be relevant and acceptable to use in patients with leprosy and other disability [20, 24], the hindrances of participation among T2DM patients with VD might be different. The tool should be modified since the limited social life in these patients is related more with the dizziness symptom caused by VD and fear of falling. Also, the concept of participation in T2DM is initially introduced in Indonesia, so the measurement tool for exploring this problem is important. Therefore, the objectives of this study were (1) to translate and cross-culturally adapt the PSSS into Indonesian, (2) to determine the measurement properties of the translated version, and (3) to determine the factors contributing to participation restriction in T2DM patients who have VD.

## 2. Methods

This study procedure consisted of the translation of the PSSS scale from English into Indonesian Bahasa version and the testing of measurement property of the Indonesian Bahasa version of the PSSS (PSSS-IB) based on the protocol recommended by Beaton et al. [27]. Furthermore, the analysis was carried out to determine the factors contributing to participation restriction. The research protocol was approved by the Institutional Review Committee, Mahidol University (MU-CIRB 2020/098.2004), and the Institutional Review Committee Medical Faculty, Muhammadiyah University of Surakarta, Indonesia (3009/B.1/KEPK-FKUMS/VII/2020). Before the data collection, the authors of the original tool gave permission for the translation and modification.

### 2.1. Participants

The participants were recruited from a community center for diabetes mellitus, organized by Prolanis Indonesia, Surakarta City, Central Java Province, Indonesia. The poster and leaflet advertising of the project were distributed to the members of the centers as well as the words of mouth to invite ones who were interested. The convenient sampling is then used with the inclusion criteria including age over 40, diagnosed with T2DM for more than 5 years, and having VD. The VD was identified by the clinical tests performed by a registered physical therapist with 8 years of clinical experiences. She was also specifically trained to perform these vestibular tests. The positive results of at least one of the three vestibular clinical tests, including the head impulses, the Dix Hallpike, and the supine roll tests, were used for confirmation of VD. The head impulse test was used to identify vestibular hypofunction by demonstrating a corrective saccade during head movement [28]. The positive Dix Hallpike test indicated the sign of posterior BPPV if the nystagmus was observed [29]. The nystagmus during the supine roll test was used to identify the horizontal BPPV [30]. The exclusion criteria were the inability to stand or walk independently, blind, having neurological problems affecting postural balance and movements, acute orthopedic problems, and not being able to follow verbal commands. After thorough explanation of the objectives and protocol, the participants who agreed to participate were asked to sign an informed consent form.

The sample size of this study was determined by the number of minimum acceptable reliability of 0.6, expected reliability of 0.8, and significance level of 0.05. Using the power of 80%, twice repetitions, and the expected dropout rate of 10% [31], the calculated number of participants was 58.

### 2.2. Instruments

The PSSS questionnaire was composed of 13 items evaluating the level of participation among patients with T2DM with VD [24]. If the participants respond as no or sometimes, the follow-up question was asked to specify the area of problem as 0: not relevant, 1: no problem, 2: small, 3: medium, or 5: large. The scores were summed to get the total level of participation. The total score was 65 with recommended interpretations of no significant restriction (0-6), mild restriction (7-13), moderate restriction (14-30), severe restriction (31-50), and extreme restriction (51-65) [32].

The glycemic level was measured by taking blood intravenously after the patient had fasted for 8 hours. The Diabetic Peripheral Neuropathy (DPN) was assessed by the physical examination according to the Michigan Neuropathy Screening Instrument (MNSI). The score ≥ 2 was considered DPN [33]. The pain at the lower extremity associated with DPN was also assessed by Numeric Rating Scale (NRS). Number of falling was recorded using the previous twelve-month timeframe [34]. The static balance was measured by the Modified Clinical Test of Sensory Interaction of Balance (mCTSIB) for 30 seconds; then the dynamic balance was measured by the Timed Up and Go test (TUG) and the Functional Gait Assessment (FGA). The TUG score more than 11.1 sec was considered high risk of falling [35], and the cut point of <22 of the FGA test was categorized as high risk of falling as well [36].

The reduction of visual function was identified by a letter chart that using in a unit from the minimum visual angle resolvable of the logarithm arranged by the distance [37]. The letter count less than 75 or 0.63 of decimals count was considered low visual acuity [38, 39]. The fear of falling was identified by the Activities Balance Confidence-16 scale (ABC-16), and score less than 67 was marked as low balance confidence [40]. The cognitive function was measured by the questionnaire of the Montreal Cognitive Assessment-Indonesian version (Moca-Ina), and score less than 26 was identified as a mild cognitive impairment [41]. The DHI score range between 0 and 100, with the points of 0-30 reflecting mild handicap, 31-60 moderate handicap, and 61-100 severe handicap [42]. The disability of dizziness was revealed by the Dizziness Handicap Inventory scale (DHI). The Five Time Sit to Stand test (FTSTS) was used to measure the lower limb muscle strength. The cut score of FTSTS test of 15 sec was identified as fall risk in individual with VD [43].

### 2.3. Study Procedure

#### 2.3.1. Phase I: Translation and Cross-Cultural Adaptation

The first translation step was undertaken by two Indonesian physiotherapists who were proficient in English and were not familiar with the PSSS. They translated the original PSSS from the English version to Indonesian Bahasa. The translated questionnaire draft was assessed whether there was equivalent context of Indonesian culture and language. The third step was the back translation into English version. This was done by two independent English-speaking translators, who never knew the PSSS and did not have medical background. This process was synthesizing and verifying the PSSS-Ina to match the sense of the original PSSS. The last process and both forward and backward translations were submitted to the committee consisting of all translators and other two experienced physiotherapists. They reviewed the original and translated versions of the PSSS to reassure the semantic, idiomatic, experiential, and conceptual equivalence. The discussion was done for each question until achieving the final agreement.

#### 2.3.2. Phase II: Psychometric Testing

**Figure 1:**
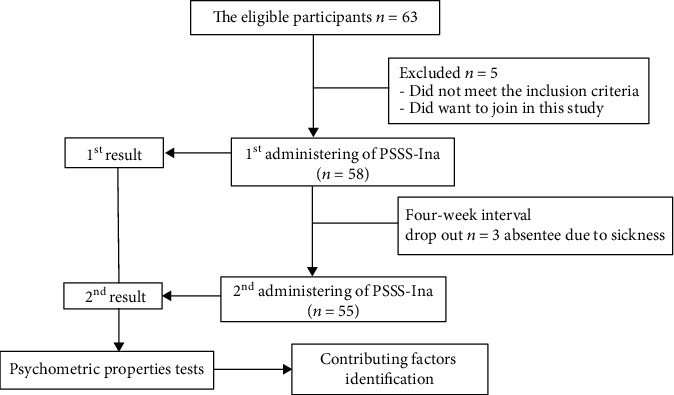
Flow diagram of the study.

In this phase, the PSSS-Ina was tried out to measure the participation level among individuals with T2DM with VD. The eligible participants who passed the inclusion and exclusion criteria answered each question of the PSSS-Ina according to their recent condition. The participants read and filled in the PSSS-Ina questionnaire by themselves at the clinical setting. However, if any participant had difficulty reading, the researcher read the questions to them. The researcher also clarified if there was any doubt about the content of the questionnaire. The retest by answering the same questionnaire again was done after four-week interval at the same setting. The flow of study protocol is presented in Figure 1.

#### 2.3.3. Phase III: Contributing Factor Identification

The factors contributing to participation restriction in T2DM patients who have VD were also determined. The potential factors considered to include in the multiple regression analysis were identified based on the literature review. Twelve factors were included, i.e., glycemic level, score of MNSI, score of NRS, number of falling, time of mCTSIB, time of TUG, score of FGA, score of visual function, score of ABC-16, score of Moca-Ina, score of DHI, and time of FTSTS test.

### 2.4. Data Analysis

The Cronbach's *α* was used to determine the internal consistency of PSSS-Ina, reflecting the homogeneity or unidimensional of the scale. The Intraclass Correlation Coefficient (ICC) of the PSSS-Ina was analyzed the test-retest reliability between two administrations of four weeks apart. The value of <0.40 was considered poor correlation, 0.40–0.59 was fair, 0.60–0.74 was good, and >0.75 was excellent [44]. Furthermore, the criteria of the correlations of corrected item and total correlation greater than 0.3 were used in the content validity analysis, confirming that all items were correlated with the total score. Floor and ceiling effects were considered, if >15% of the participants achieved the lowest or highest possible total score, respectively [31]. The standard error of measurement was also used to confirm the test reliability. Meanwhile, the minimum detectable change was at 95% confidence level (MDC_95_) for the PSSS-Ina.

A principal component analysis with varimax rotation was performed to examine the component structure of the 13-item the PSSS-Ina. All eigenvalues, which were greater than 1.0, were plotted on a Cattell's Scree plot, and the items with loadings above 0.40 were assumed to load on a given factor. The Kaiser–Meyer–Olkin measurement of sampling adequacy (KMO) and Bartlett's test were calculated to confirm that the sample was large enough to perform a satisfactory factor analysis [45].

The multiple logistic regression model was constructed to identify the variables contributing to the PSSS-Ina score. The correlation between PSSS-Ina and the predictor variables are identified by the Spearman Rank test. The estimated coefficients and their standard errors (SEs) were calculated using the method of maximum likelihood. The entry method was used for variable selection. The calibration was assessed using the Hosmer-Lemeshow goodness-of-fit test [46]. Discrimination was assessed using the area under the receiver operating characteristic (AUROC) [47] to evaluate overall predictive accuracy of the model.

## 3. Results

The PSSS-Ina was administered to 58 adults with T2DM with vertigo complaint and positive VD test. There were three participants who did not complete the second administration. The demographic and clinical characteristics of the sample are summarized in Table 1. All participants in this study had positive results of at least one of three VD screening test. The posterior BPPV indicated by positive Dix Hallpike test was presented in more than half of participants. They also had DPN (41.8%) and impaired visual acuity (34.5%). More than half of participants were identified as having a high risk of falling by the TUG test, FGA test, ABC-16 scale, and FTSTS test. The mild cognitive impairment was also prevalent in this study.

The translated PSSS-Ina, consisting of 13 items, was judged by the expert panel on the relevance and phrasing of the instrument items. The experts had suggested the possible improvements for each item in phrasing. Two items, i.e., item 5 and item 6, were modified to be culturally applicable to the T2DM patients with VD in Indonesia. For item 5, the original version of “Do you take part in major festivals and rituals as your peers do? (e.g. weddings, funerals, religious festivals)” was adapted to “Compared to other people, do you take part in community events? (e.g. weddings, funerals, religious festivals).” The original question of item 6 of “Do you take part in social activities as much your peers do? (e.g. in sports, chat, meetings, religious or community activities)” was modified to “Compared to other people, do you take part in social activities? (e.g. in sport, routine meeting, religious meeting).” Similar with other studies [20, 24], the word “peer” was also confusing for the participants. So it was changed into “compared to other people” for all items.

As presented in Table 2, the good internal consistency of PSSS-Ina was confirmed. Cronbach's alpha of the PSSS-Ina is 0.84. If an item was deleted, the value of Cronbach's alpha for all items in each scale was still higher than 0.8. The mean of PSSS-Ina total score was 32.11 ± 6.3. The test–retest reliability between two measurement sessions with four-week duration was excellent (ICC of 0.93, *p* < 0.001, and 95% CI: 0.88–0.95). The correlation coefficient between two administrations by product-moment was high (*r* = 0.88). In addition, the values of the corrected item-total correlation for all items and total score were also confirmed as the values above 0.3 (range: 0.35-0.84). No floor and ceiling effects for the PSSS-Ina were found in the T2DM with VD. The maximum and minimum scores were 52 and 20 points, respectively, with 1.8% of participants (*n* = 1) reaching these scores. Meanwhile, the standard error of measurement of the PSSS-Ina was 1.66. The MDC_95_ for the PSSS-Ina total score was 4.57, representing the required amount of change in patient status to exceed chance variation.

**Figure 2:**
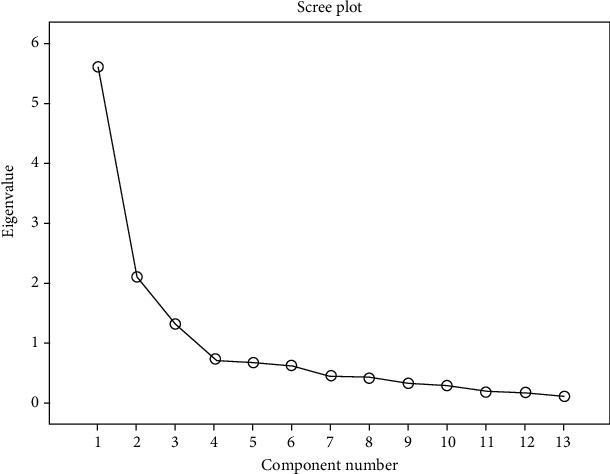
The Scree plot of items of the PSSS-Ina (components with high eigenvalues, >1.0).

For factor analysis, the criteria needed to be satisfied were checked for the adequacy of the exploratory factor analysis. Bartlett's test of sphericity was significant (*X*^2^ = 590.398, *p* < 0.001), and the KMO measure of sampling adequacy was acceptable (0.683). The anti-image matrices and the communalities were more than 0.50. These established the sampling adequacy of the items in PSSS-Ina. The components with eigenvalues greater than 1.0 produced three factors. The Scree plots of items of the PSSS-Ina showed three components matrix (Figure 2). Also, the absolute values of factor loadings with many item correlation coefficients were above the threshold of 0.40 (Table 3). The first component factor accounts for the maximum part of the variance of 41.8% with eigenvalue 5.4. Factor 1 comprises 6 items, including items 6, 7, 8, 11, 12, and 13. The second component explains the maximum part of the remaining variance of 20.5% with an eigenvalue of 2.6. On this factor, loadings are found among six items including items 2, 3, 5, 7, 8, and 9. The third factor explains 10.6% of the variance with an eigenvalue of 1.3 and confirms four items including items 1, 4, 9, and 10.

**Figure 3:**
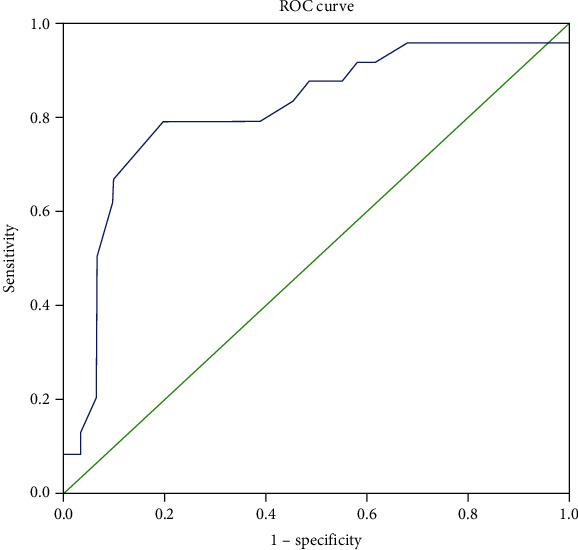
Area under receiver operating characteristic curve (AUROC) for participation restriction used cut of 31 of PSSS-Ina.

As shown in Table 4, the mCTSIB and lower limb muscle strength were significantly correlated with the PSSS-Ina.  Table 5 shows the final model of logistic regression. The Hosmer and Lemeshow test of multiple logistic regression revealed the final model with a *p* value of 0.442. Using criteria of severe restriction with the cut off score of 31, the mCTSIB was the factor contributing to the PSSS-Ina score (Adj. OR 1.089; 95% CI =1.029 to 1.153, *p* = 0.003) and *R*^2^ = 0.362. The area under receiver operating characteristic (AUROC) (Figure 3) for participation severe restriction was 0.815 (Table 6).

## 4. Discussion

The PSSS is successfully translated and cross culturally adapted into Indonesian, PSSS-Ina, using the recommended guidelines [27]. Regarding the content, minor adaptions such as changing of the measurement units to metric and words or phrases such as dress items to commonly used equivalent Indonesian terms are commenced for the comprehensibility. As observed when testing the translated questionnaire, participants often compared themselves with a friend with similar context. Some questions could not be phrased as appeared in the questionnaire, but the interviewer had to clarify the meaning of the questions and sometime using examples. After adjusting these questions, the content of all items was adequately applicable.

The PSSS-Ina demonstrates a good internal consistency for total score and each item, as presented in Table 2. The test–retest reliability quantified by ICC shows that the ICC is higher than 0.75, indicating “excellent” reliability, and has high correlation coefficient between two administrations. Notably, this ICCs value is considerably higher than the study of previous Indonesian translated version applied in the patients with leprosy [24].

The values of correlation of each item and the total score were also confirmed above 0.3 for all items. Items 6 and 12 had the lowest value, and item 9 had the highest correlation. Item 6 asked about “take part in social activities.” Moreover, we had replaced the word “chat and meeting” with “routine meeting in your village” and gave the examples (as activities program at village level to educate people on various aspects of family welfare) to make the questions more understandable. Unfortunately, the item validity is still low. Item 12 regarding the opinion account in family also had low corrected item-total correlation. This might indicate the different context of this issue relative to other questions in the questionnaire. Items 1 and 4 appeared to have comparable levels of correlation with the total score. Item 1 (find work) was not applicable for some respondents, especially the ones who were the housewives. However, after explaining and comparing themselves with other housewives, the respondents could give a response. Similarly, for item 4, visiting outside the village was rather difficult for these patients. They appeared to have limitation for activities because of their characteristics of having dizziness symptom, averaged age over 60 years, and most of them were a housewife or male retiree. This might be in accordance with the cultural context for older people in Indonesia to be more comfortable staying with family at home.

The exploratory factor analysis in our study showed three factors extracted from the PSSS-Ina scale. The factors of the PSSS-Ina could be described as activity participation, social engagement, and work-related participation. Factor 1, the activity participation, referred to the execution of physical and social activities [48] and was mainly performance-oriented participation [24]. Factor 2, the social engagement, was the togetherness-oriented participation focused on performing meaningful social roles [49]. Factor 3 involved the work-related participation. Items 7, 8, and 9 were loaded in two factors, suggesting that the differentiation between factors might be difficult in some circumstances. The experts also described the difficulty to distinguish the social participations and the social engagement. These terms were used inconsistently in the literature and could sometimes be confusing with several similar but distinct concepts of social sciences [50]. In contrast, a previous study of the original P-scale showed a two-factor model, consisting of “work-related participation” and “general participation” (32). However, another study reported three factors encompassed the principal constructs related to disability and participation after adding items of related to work and gainful employment [51].

Furthermore, the original P-scale was reported to be difficult to administer and interpret [20] while the shortened and simplified version, the PSSS needed less time for response and having more understandable questions [20]. In this study, the issue of administering the PSSS-Ina was still found since the shorter question structure of the simplified scale was confusing for some respondents. However, the researchers found that with the cultural adaptation and few explanations and examples during administration, the participants could response to the questionnaire handily.

The T2DM patients were reported to have limited social engagement [52] and might be partly associated with the complications of VD. The impact of VD symptoms itself has been clearly known to restrict the participations, such as travelling, getting together with the community, having fun, and else to avoid the fear of provoking the dizziness or vertigo symptoms [53]. However, the participation restriction specifically in T2DM patients with VD is not much explored recently. The impact of VD symptoms in older adults is clinically significant because they are one of the ten main reasons leading patients to seek medical attention at the emergency room [54]. The VD symptoms were also the predictors of diminished balance and fall [8], reduced self-confidence, increased depression and frustration, having major impacts in falls, prolonged sick leave, morbidity, and associated low quality of life individuals [55, 56].

The correlation analysis showed that mCTSIB and lower limb muscle strength were significantly correlated with the PSSS-Ina. The final model of multiple logistic regression revealed that the mCTSIB as the only factor predicting the participation restriction used cut of 31 of the PSSS-Ina with 26% variance accounted and 80% prediction accuracy. The mCTSIB was used to determine the ability to maintain balance in different conditions of sensory dependency. Specifically, in condition 4, stand on foam and eyes closed could determine the function of vestibular system on balance performance [57]. Therefore, the results of this test could determine the severe participation restriction in participants with positive vestibular dysfunction test. The patients with vestibular disorder usually have poor balance, high risk to fall, and fear of falling which consequently impacting the level of social participation [7, 53].

This study had some limitations. The PSSS-Ina was tested for its internal consistency, test–retest reliability, factor confirmation, and predicting factors of severe level scoring. The construct and criterion validity as well as the responsiveness of the scale were not addressed. Further studies focusing on psychometric testing of the validity and the responsiveness are still needed. Even the KMO was calculated to confirm sampling adequacy but one should be cautious that with the larger sample size, the factor structure might differ from the results shown in this current study. Moreover, since the study was conducted in the community setting using the clinical screening tests to confirm the VD, the VD diagnosis might be inconclusive. A further study in patients with VD confirmed by the standard tests like Vestibular-evoked myogenic potentials (VEMPs) test or caloric tests is therefore recommended.

## 5. Conclusion

The Indonesian version of the PSSS-Ina demonstrates excellent comprehensibility and reliability. Therefore, the tool is applicable for patients with dizziness or vertigo symptom who might have significantly reduction of social participation for the comprehensive management. This scale is therefore recommended for assessing the T2DM patients with VD in both clinical practice and research settings.

## Figures and Tables

**Table 1:** Demographic and clinical characteristics of participants.

Variables	*N* (%)	Min	Max	Mean ± SD
Age (years)		48	78	62.1 ± 7.1
Gender				
Male	22 (40%)			
Female	33 (60%)			
Number of falling within a year		0	3	1.3 ± 0.6
No fall	42 (76.4%)			
1 fall	10 (18.2%)			
2 or more fall	3 (5.5%)			
DM duration (years)		5	20	7.3 ± 3.9
5–10	34 (61.8%)			
>10	21 (38.2%)			
Hypertension				
Yes	27 (47.1%)			
No	28 (50.9%)			
Body Mass Index		20.42	32.03	24.9 ± 2.5
Underweight	1 (1.8%)			
Normal	21 (38.2%)			
Overweight	31 (56.4%)			
Obesity	55 (3.6%)			
Fasting glycemic level (FGL) (mg/dl)		90	349	164.3 ± 50
Pain in lower extremity		2	10	5.76 ± 1.2
Michigan Neuropathy Screening Instrument (MNSI) (score)		0	4	1.6 ± 1.1
Positive diabetic peripheral neuropathy	23 (41.8%)			
Negative diabetic peripheral neuropathy	32 (58.2%)			
Visual acuity (score)		0.4	1.0	0.7 ± 02
Low visual acuity	19 (34.5%)			
Modified clinical test of sensory interaction in balance (mCTSIB) (secs)		51.6	108	81.5 ± 15
Five Time Sit to Stand test (FTSTS) (secs)		10.6	28.2	16.2 ± 3.4
High risk of falling	39 (70.9%)			
Low risk of falling	16 (29.1%)			
Timed Up and Go test (TUG) (secs)		10.12	24.6	15.7 ± 2.8
High risk of falling	54 (98.2%)			
Low risk of falling	1 (1.8%)			
Functional Gait Assessment (FGA)		14	26	19.5 ± 3.3
High risk of falling	38 (69.1%)			
Low risk of falling	17 (30.9%)			
Activities Balance Confidence-16 (ABC-16) (%)		52.5	71.2	63.8 ± 5.2
High risk of falling	37 (67.3%)			
Low risk of falling	18 (32.7%)			
Dizziness Handicap Inventory (DHI) (score)		50	90	69.3 ± 10.1
Moderate handicap	12 (21.8%)			
Severe handicap	43 (78.2%)			
Montreal Cognitive Assessment (Moca)		14	28	21.7 ± 3.9
Normal cognitive	14 (25.5%)			
Mild cognitive impairment	43 (78.2%)			
Participation Scale Short Simplified Indonesian version (PSSS-Ina) (score)		20	48	32.1 ± 6.3
Moderate restriction	24 (43.6%)			
Severe restriction	31 (56.4%)			
Positive vestibular screening tests				
Head impulse test	15 (27.3%)			
Dix Hallpike test	32 (58.2%)			
Supine roll test	8 (14.5%)			

**Table 2:** The item-total correlations of the PSSS-Ina.

	Corrected item-total correlation	Cronbach's alpha if item was deleted
(1) Have opportunities to find work	.490	.845
(2) Work hard as others	.805	.826
(3) Contribute to household economically	.823	.819
(4) Visits outside village/neighborhood	.418	.849
(5) Take part in community events	.659	.831
(6) Take part in social activities	.350	.847
(7) Have same respect in community	.694	.831
(8) Visit other people in the community	.663	.833
(9) Move around inside & outside the house	.849	.814
(10) Visit public places	.665	.831
(11) Do household work	.570	.843
(12) Opinion account in family discussion	.360	.849
(13) Comfortable meeting new people	.515	.841

Cronbach's alpha = 0.84; the PSSS-IB scale.

**Table 3:** Principal component factor analysis with varimax rotation for individual items.

Items	Factor loadings		
	1	2	3
(1) Compared to other people, how easy is it for you to find work?			.864
(2) Compared to other people, do you work hard (same hours, type of work, etc.)		.889	
(3) Compared to other people, do you contribute to the household economically?		.891	
(4) Compared to other people, do you visit outside your village/neighborhood? (except for treatment; e.g., bazaars and markets)			.925
(5) Compared to other people, do you take part in community events (e.g., weddings, funerals, and religious events)		.723	
(6) Compared to other people, do you take part in social activities? (e.g., in sports, routine meeting, and religious or community activities)	.847		
(7) Compared to other people, do you have the same respect in the community?	.530	.627	
(8) Compared to other people, do you visit other people in the community?	.571	.561	
(9) Compared to other people, do you move around, inside and outside the house, and around the village or neighborhood?		.617	.559
(10) In your village/neighborhood, do you visit public places as often as other people (e.g., schools, shops, offices, market, and tea/coffee shops)?			.646
(11) In your home, do you do household work?	.847		
(12) In family discussions, does your opinion count?	.900		
(13) Are you comfortable meeting new people?	.701		

Extraction method: principal component analysis. Rotation method: varimax with Kaiser normalization. Rotation converged in 5 iterations.

**Table 4:** The correlation between PSSS-Ina and predictor variables in T2DM with vertigo complaint and positive VD tests.

Independent factor	Predictor variables	*p* value
PSSS-IB	Gender	.352
	Age	.902
	Fasting glycemic level	.741
	Hypertension status	.493
	Pain	.600
	Number of falling	.066
	BMI	.186
	Education	.958
	Duration of DM	.279
	mCTSIB	<.001∗
	TUG	.096
	FGA	.155
	Lower limb muscle strength	.025∗
	Visual acuity	.271
	Fear of falling	.115
	DHI	.288
	Cognitive function	.206
	DPN	.098

^∗^Significant value < 0.05.

**Table 5:** The final model of multiple logistic regression of PSSS-Ina used cut of 31 as severe restriction in T2DM with vertigo complaint and positive VD tests (*n* = 55).

Variable independent	Predictor variables	b	*p-*value	Adjusted OR (95% CI of OR)
PSSS-Ina (31)	mCTSIB	.085	.003∗	1.089 (1.029, 1.153)
	Lower limb muscle strength	-.049	.698	.953 (.746, 1.217)
	Constant	-6.531	.083	.001

^∗^Significant value < 0.05.

**Table 6:** Area under receiver operating characteristic curve (AUROC) for participation restriction used cut of 31 of PSSS-Ina and maximum likelihood of mCTSIB.

Variables	AUROC	SE	*p* value	95% CI	
				Lower	Upper
PSSS-IB	.815	.065	<0.001	.692	.937

## Data Availability

The data used to support the findings of this study are available from the corresponding author upon request.
